# Split Thickness Skin Grafting in Patients with Stable Vitiligo

**DOI:** 10.4103/0974-2077.79189

**Published:** 2011

**Authors:** Farah Sameem, Sheikh Javeed Sultan, Qazi Masood Ahmad

**Affiliations:** *Department of Dermatology, STD and Leprosy, Government Medical College, Srinagar, Kashmir, Jammu and Kashmir, India*

**Keywords:** Repigmentation:, split thickness skin grafting, vitiligo

## Abstract

**Background:**

Vitiligo is an acquired disorder of depigmentation. Various surgical modalities are recommended for quicker resolution of lesions in stable cases.

**Aim:**

To report the efficacy of split thickness skin grafting in a series of 50 patients with stable vitiligo.

**Materials and Methods:**

Fifty patients with stable vitiligo, not responding to adequate trial of medical line of treatment were taken in this prospective study. After doing complete investigations, including coagulogram, they were subjected to split thickness skin grafting.

**Result:**

Satisfactory cosmetic results were obtained in all cases. Colour match was good with minimal complications reported.

**Conclusion:**

Split thickness skin grafting remains a promising option for patients with stable recalcitrant vitiligo.

**Limitations:**

Comparison of efficacy and side effects of various vitiligo surgical modalities was not done.

## INTRODUCTION

Vitiligo is a pigmentary disorder characterized by areas of depigmented skin resulting from loss of functioning epidermal melanocytes and sometimes hair follicle melanocytes. It affects between 1% and 2% of the general population without any racial, sexual, or regional differences in prevalence. Though vitiligo is not a life-threatening disease, however, the psychosocial impact of the disease is devastating, particularly in darker skins. Onset may occur at any age, but the incidence usually peaks in the second and third decades of life. Taking the extent and distribution into consideration, vitiligo is categorized as generalized, focal, acrofacial, segmental, and universal.[1–4]

Both medical and surgical therapeutic approaches can be used effectively in the management of vitiligo. The surgical methods are recommended for lesions that are stable and refractory to medical therapies. The surgical techniques are based on the basic principle of restoring melanocytes in recipient vitiliginous sites, obtained from pigmented donor skin. There are several methods of melanocyte transplantation, such as suction blister grafting, split thickness skin grafting (STSG), minigrafting (punch grafting), follicular grafting, cultured-melanocyte transplantation, and noncultured-melanocyte transplantation.[5–8] Herein we report our experience regarding STSG in a series of 50 vitiligo patients.

## MATERIALS AND METHODS

This study was carried out amongst 50 vitiligo patients attending the out-patient clinic during Jan 2006 and Dec 2009. The criteria for selection was that the patients’ disease should be stable for more than 2 years, stability being defined as no eruption of fresh lesions and no extension of pre-existing lesions. Patients having a stable disease and not responding to medical line of management were included in the study. Patients with an active disease, keloidal tendency, active infection, and altered texture of vitiligo patches were not taken up. Baseline investigations included complete haemogram and coagulogram, HIV, and Hepatitis B and C virus serology. Any associated medical condition was ruled out or appropriately managed. Realistic expectations on part of the patients were emphasized. After taking an informed consent, premedication and preparation, the recipient site was draped and dermabraded by mechanical Maneksha’s dermabrader. The donor site for graft included anterior, lateral, or posterior thigh or the upper arms.

The donor graft was taken by means of Humby’s or Slivers knife. After needling the graft, it was placed on the dermabraded bed and fixed by surgical glue. A composite dressing was placed at both sites. The patient was put on antibiotics and anti-inflammatory drugs for 1 week. Antiseptic dressing was done on 2^nd^ day and then on 10^th^ day. At the first dressing, signs of seroma, graft displacement, or hematoma formation was checked.

Postoperatively, the patients were put on topical psoralens with UVA therapy. A short course of corticosteroids at the dose of 30 mg prednisolone/day was given with a gradual taper over 4 weeks, at the end of 6 weeks (in 10 cases) in patients with slow or no response at that time period.

## RESULTS

Fifty patients with stable vitiligo were taken up for STSG. These included 37 females and 13 males. The age range varied from 12 to 45 years [Table 1]. The duration of disease ranged from 3 to 15 years. The various clinical variants and the sites taken up for grafting are shown in Tables 2 and 3. The average number of sittings required were 2–3. The interval between 2 sittings at the same site was fixed at 6 months, whereas adjacent lesions could be taken up after 3 weeks. The size of lesions ranged from 2to 6 cm^2^. The shape of lesions was amoeboid, segmental, or circular.

**Table 1:** Age distribution of patients

Age (years)	<20	20–30	>30
Male	01	10	02
Female	04	28	05

**Table 2:** Clinical variants of vitiligo

Clinical variants	No. of patients
Vitiligo vulgaris	4
Focal	15
Segmental	23
Acrofacial	8
Total	50

**Table 3:** Sites taken up for grafting

Site grafted	No.
Face	17
Arm	8
Leg	6
Neck	15
Lips	3
Areola	1

The results of the procedure were noted in the form of intensity of pigment, perigraft spread of pigment, and the color match with the surrounding skin. At the same time, the texture of skin over the grafted area was matched with that of the surrounding normal skin. Perigraft spread of pigment was the best for facial lesions and lesions close to hair line and the poorest for ankle lesions.

**Figure 1:**
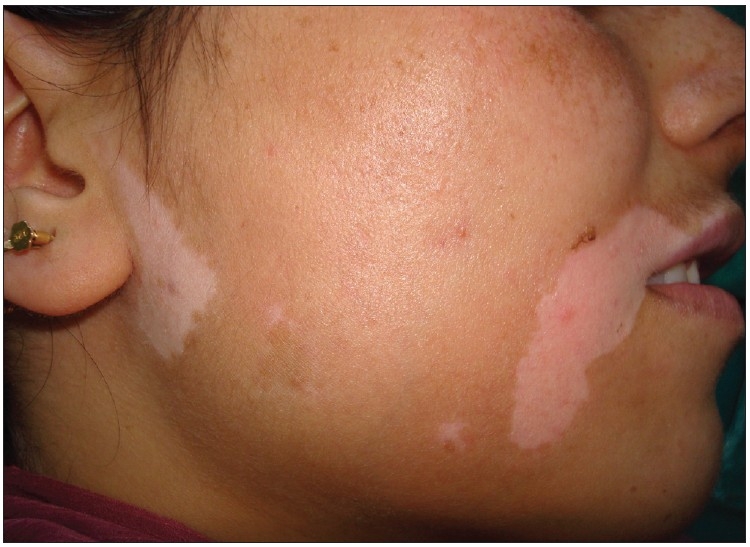
Preoperative picture of vitiligo lesions on face

**Figure 2:**
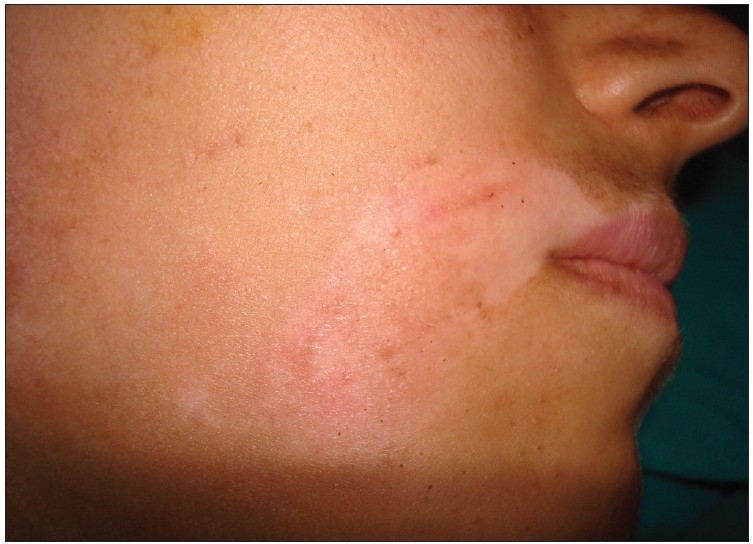
Six weeks postoperative picture showing moderate improvement

Results were better in younger patients. Facial lesions responded most satisfactorily [Figures 1 and 2]. Amongst the various clinical variants, focal and segmental vitiligo responded the best, acral vitiligo showed a lesser response vis-a-vis intensity of color and its spread. Patients with a shorter duration of the disease had a more satisfactory response. Individuals with darker skin type and more pigmented hairs had an earlier and better response as compared with fair complexioned ones. The thickness of the graft was a defining factor for the satisfactory response. Thinner grafts that were shed off completely after donating the melanocytes to vitiliginous areas were the best ones. Thicker grafts would result in a stuck on appearance at recipient site and infection and scarring at donor site. There were 3 cases of graft displacement, especially for mobile areas and neck. Secondary infection of the donor or recipient site was seen in 2 cases. Donor site depigmentation developed in one patient with reactivity of vitiligo. The earliest response was seen at 4 weeks. Complete response took up to 6 months.

Postoperative topical psoralens with UVA was seen to produce better colour intensity and spread of pigment, thus hastening the complete response stage. Postoperative steroids were found to enhance the pigmentation and shorten the stage to complete response in patients who showed no pigment at the end of 6 weeks.

## DISCUSSION

Vitiligo is a socially, emotionally, and cosmetically disfiguring entity for which patient is desperate to get as quick a response as possible. With the advent of various surgical modalities a ray of hope can be offered to these patients, especially those not responding to medical therapy. Depigmentation in vitiligo is the result of depletion of melanocytes following their destruction by the underlying disease.[1–3] The various surgical techniques are based on the basic principle of restoring melanocytes on vitiliginous sites from pigmented donor skin.[5,6]

For better colour and texture match, faster resultsand the highest success mean rate, split thickness skin grafting (STSG) has achieved pre-eminence. This involves transfer of epidermis and the uppermost part of superficial dermis to achieve transfer of melanocytes and keratinocytes from donor graft to the underlying dermabraded vitiliginous area. It is possible that growth factors and cytokines released during wound healing help in migration, transplantation, and multiplication of melanocytes.[9–12]

Our study revealed better results for facial lesions, especially in younger patients with dark skin, shorter duration of disease, and segmental type of vitiligo. As there is a risk of graft displacement on the neck and other mobile areas, longitudinal strips of grafts arranged parallel to the skin creases can be used at these sites. Furthermore, postoperative use of corticosteroids and topical psoralens with UVA, were found to enhance the pigmentation and reduce the time of appearance of complete response. The main drawback of this procedure is that expertise is required for taking a thin graft. However, in properly selected patients with good operator expertise, STSG was found to be a satisfactory tool for reducing patient morbidity and improving the quality of life in our patients with vitiligo, as can be judged from the enthusiastic patient response that we continue to get.
